# Inhibition of early T cell cytokine production by arsenic trioxide occurs independently of Nrf2

**DOI:** 10.1371/journal.pone.0185579

**Published:** 2017-10-19

**Authors:** Kelly R. VanDenBerg, Robert A. Freeborn, Sheng Liu, Rebekah C. Kennedy, Joseph W. Zagorski, Cheryl E. Rockwell

**Affiliations:** 1 Department of Pharmacology & Toxicology, Michigan State University, East Lansing, Michigan, United States of America; 2 Institute for Integrative Toxicology, Michigan State University, East Lansing, Michigan, United States of America; 3 Cell and Molecular Biology Program, Michigan State University, East Lansing, Michigan, United States of America; Universite Paris-Sud, FRANCE

## Abstract

Nuclear factor erythroid 2-related factor 2 (Nrf2) is a stress-activated transcription factor that induces a variety of cytoprotective genes. Nrf2 also mediates immunosuppressive effects in multiple inflammatory models. Upon activation, Nrf2 dissociates from its repressor protein, Keap1, and translocates to the nucleus where it induces Nrf2 target genes. The Nrf2-Keap1 interaction is disrupted by the environmental toxicant and chemotherapeutic agent arsenic trioxide (ATO). The purpose of the present study was to determine the effects of ATO on early events of T cell activation and the role of Nrf2 in those effects. The Nrf2 target genes Hmox-1, Nqo-1, and Gclc were all upregulated by ATO (1–2 μM) in splenocytes derived from wild-type, but not Nrf2-null, mice, suggesting that Nrf2 is activated by ATO in splenocytes. ATO also inhibited IFNγ, IL-2, and GM-CSF mRNA and protein production in wild-type splenocytes activated with the T cell activator, anti-CD3/anti-CD28. However, ATO also decreased production of these cytokines in activated splenocytes from Nrf2-null mice, suggesting the inhibition is independent of Nrf2. Interestingly, ATO inhibited TNFα protein secretion, but not mRNA expression, in activated splenocytes suggesting the inhibition is due to post-transcriptional modification. In addition, c-Fos DNA binding was significantly diminished by ATO in wild-type and Nrf2-null splenocytes activated with anti-CD3/anti-CD28, consistent with the observed inhibition of cytokine production by ATO. Collectively, this study suggests that although ATO activates Nrf2 in splenocytes, inhibition of early T cell cytokine production by ATO occurs independently of Nrf2 and may instead be due to impaired AP-1 DNA binding.

## Introduction

Inorganic arsenic is an environmental contaminant and is also used clinically as a chemotherapeutic in the form of arsenic trioxide. As an environmental contaminant, it is often found in water sources and has been found to elicit numerous toxic effects. It is present in two valence states, arsenite (As^3+^) or arsenate (As^5+^), with arsenite considered to be the most toxic form [1,2]. Exposure to inorganic arsenic has been linked to an increased incidence of gastroenteritis, cardiovascular disease, diabetes, and various forms of cancer [1,3–5]. Other evidence also suggests chronic exposure to arsenic causes liver injury, immunotoxicity, peripheral neuropathy and other neurotoxic effects. The toxic effects of arsenic are likely mediated through multiple mechanisms, including induction of oxidative stress, alteration of various sulfhydryl-containing proteins, disruption of mitochondria energy generation, and DNA adducts, among others.

Numerous studies have shown that chronic exposure to inorganic arsenic impairs the human immune response [6,7]. Arsenite alters the abundance of particular immune cell populations—such as those of eosinophils and monocytes—and modulates expression of genes associated with immune response in peripheral blood lymphocytes [8,9]. Of relevance to the present studies, arsenic has been shown to inhibit events associated with early T cell activation, including T cell proliferation, as well as early cytokine secretion [10]. These effects of arsenic can ultimately impact functional immunity [7,11,12]. In addition, arsenic impairs immunosurveillance, which may ultimately contribute to its carcinogenicity [7,12]. However, the mechanism through which arsenic affects these immune responses is not fully understood.

Inorganic arsenic activates the transcription factor nuclear factor erythroid 2-related factor 2 (Nrf2) in a variety of different cell types, including osteoblasts, keratinocytes, and bladder epithelial cells, among others [13–15]. Nrf2 is a stress-activated transcription factor that is responsible for inducing a battery of cytoprotective genes [16,17]. Under homeostatic conditions, Nrf2 is sequestered in the cytoplasm and degraded by the proteasome due to association with its repressor protein Kelch-like ECH-associated protein 1, Keap1 [18,19]. Under stressful conditions, such as those induced by oxidative or electrophilic stimuli, Nrf2 is no longer degraded by the proteasome and consequently translocates to the nucleus to induce numerous genes involved in antioxidative responses, xenobiotic detoxification, and glutathione homeostasis [20–22]. In murine leukocytes, Nrf2 upregulates genes such as heme oxygenase-1 (Hmox1), NAD(P)H quinone oxidoreductase-1 (Nqo1), glutamate-cysteine ligase catalytic subunit (Gclc), and others [23,24]. As such, these genes serve as useful markers for Nrf2 activation in immune cells.

In addition to its cytoprotective capabilities, Nrf2 also plays an important role in modulating immune responses. Nrf2 deficiency is associated with worsened inflammation in a number of different models of inflammatory disease, including sepsis, experimental autoimmune encephalomyelitis (a model of multiple sclerosis), autoimmune hepatitis, and airway inflammation [25–28]. An anti-inflammatory role for Nrf2 is further supported by the development of multi-organ autoimmune disease in Nrf2-null mice that resembles systemic lupus erythematosus (SLE) in humans [29,30]. Furthermore, our previous studies have shown that the Nrf2 signaling pathway modulates Th1/Th2 differentiation and early events of T cell activation in both mice and humans [31–33].

Because published studies suggest arsenic trioxide (ATO) may affect T cell function, the purpose of the present study was to determine the effects of ATO on the early events of T cell activation in anti-CD3/anti-CD28-activated murine splenocytes, specifically regarding the potential role of Nrf2 in these events.

## Materials and methods

### Materials

Arsenic(III) oxide (arsenic trioxide, ATO) was purchased from Sigma-Aldrich (St. Louis, MO), along with all other reagents, unless otherwise stated.

### Nrf2-null mice

Nrf2-null mice on a mixed C57BL/6 and AKR background were generated as previously described and were a generous gift from Dr. Jefferson Chan [16]. The mice were subsequently backcrossed 8 generations onto the C57BL/6 background, were 99% congenic (analysis performed by Jackson Laboratories, Bar Harbor, ME) and their female progeny were used for these studies. Age-matched female wild-type (C57BL/6) control mice were purchased from Charles River Laboratories (Wilmington, MA). Food and water were provided *ad libitum*. All animal studies were conducted in accordance with the Guide for Care and Use of Animals as adopted by the National Institutes of Health, and were approved by the Institutional Animal Care and Use Committee at Michigan State University.

### Cell culture

Splenocytes from C57BL/6 (wild-type) mice and Nrf2-null mice were isolated and cultured at 5x10^6^ cells/mL in DMEM media supplemented with 10% fetal bovine serum (Biowest LLC, Kansas City, MO), 25 mM HEPES, 50 μM 2-mercaptoethanol, nonessential amino acids (1X final concentration from 100X stock solution), 100 U/mL penicillin, and 100 μg/mL streptomycin. Cells were left untreated or treated with a vehicle, phosphate-buffered saline (PBS), 1 μM ATO, or 2 μM ATO for 30 min. Samples were subsequently activated with 1.5 μg/mL anti-CD3 (clone eBio500A2, Affymetrix/eBioscience, San Diego, CA) crosslinked with 1.5 μg/mL anti-CD28 (clone 37.51, Affymetrix/eBioscience) by an F(ab’)_2_ fragment species for anti-Syrian hamster IgG (Jackson ImmunoResearch, West Grove,PA) or left unactivated (BKG) for varying time-points.

### CD4^+^ T cell isolation and culture

Splenocytes were isolated as above. CD4^+^ T cells were isolated by negative selection using a commercially available kit for magnetic separation (Miltenyi Biotec, Auburn, CA). Isolated T cells were cultured at 5 x 10^6^ cells/mL in DMEM media supplemented with 10% fetal bovine serum (Biowest LLC, Kansas City, MO), 25 mM HEPES, 50 μM 2-mercaptoethanol, nonessential amino acids (1X final concentration from 100X stock solution), 100 U/mL penicillin, and 100 μg/mL streptomycin. The isolated CD4^+^ T cells were left untreated (BKG) or treated with vehicle (PBS), 0.1 μM arsenic trioxide, or 0.5 μM arsenic trioxide for 30 min. Cells were then activated with 1.5 μg/mL anti-CD3 (clone eBio500A2, Affymetrix/eBioscience, San Diego, CA) crosslinked with 1.5 μg/mL anti-CD28 (clone 37.51, Affymetrix/eBioscience) by an F(ab’)_2_ fragment for anti-Syrian hamster IgG (Jackson ImmunoResearch, West Grove,PA) or left unactivated (BKG) for 24 h.

### mRNA quantification: RT-PCR

6 h post-activation, cells were collected and total RNA was isolated via TRIzol Reagent per the manufacturer’s protocol (Life Technologies, Grand Island, NY). Isolated RNA was quantified by the NanoDrop 2000c spectrophotometer (Thermo Fisher Scientific, Waltham, MA, USA). For reverse transcription, 5 μL of diluted RNA (50 ng/μL) was added to 7.5 μL of reverse transcription master mix, which was prepared as follows: for each sample, 4 μL nuclease-free water, 2.5 μL M-MLV 5X RT Buffer (Promega, Madison, WI), 0.5 μL 10 mM dNTPs (Promega, Madison, WI), 0.16 μL random primers (Promega, Madison, WI), 0.16 μL rRNAsin (Promega, Madison, WI), and 0.16 μL M-MLV Reverse Transcriptase (Promega, Madison, WI). After reverse transcription, real-time PCR SYBR green analysis was performed to quantify IL-2, IFNγ, TNFα, GM-CSF, CD25, CD69, Hmox-1, Nqo1, and Gclc mRNA expression. Ribosomal protein L13A (RPL13A) served as the endogenous control and relative mRNA expression was calculated using the ΔΔCt method. The primers (Integrated DNA Technologies, Coralville, IA) were as follows: RPL13A forward (5’-GTT GAT GCC TTC ACA GCG TA-3’), RPL13A reverse (5’-AGA TGG CGG AGG TGC AG-3’), IL-2 forward (5’-GTC AAA TCC AGA ACA TGC CG-3’), IL-2 reverse (5’-AAC CTG AAA CTC CCC AGG AT-3’), IFNγ forward (5’-TGA GCT CAT TGA ATG CTT GG-3’), IFNγ reverse (5’-ACA GCA AGG CGA AAA AGG AT-3’), TNF-α forward (5’-ATG AGA GGG AGG CCA TTT G-3’), TNF-α reverse (5’-CAG CCT CTT CTC ATT CCT GC-3’), GM-CSF forward (5’-CCG TAG ACC CTG CTC GAA TA-3’), GM-CSF reverse (5’-TGC CTG TCA CAT TGA ATG AA-3’), CD25 forward (5’-AGG GGG CTT TGA ATG TG-3’), CD25 reverse (5’-TTG CTG ATG TTG GGG TTT CT-3’), CD69 forward (5’-AGA GAG GGC AGA AGG ACC AT-3’), CD69 reverse (5’-AAG GAC GTG ATG AGG ACC AC-3’), Hmox-1 forward (5’-GAG CAG AAC CAG CCT GAA CTA-3’), Hmox-1 reverse (5’-GGT ACA AGG AAG CCA TCA CCA-3’), Nqo1 forward (5’AAC GGG AAG ATG TGG AGA TG-3’), Nqo1 reverse (5’-CGC AGT AGA TGC CAG TCA AA-3’), Gclc forward (5’-GCA CGG CAT CCT CCA GTT CCT-3’), and Gclc reverse (5’-TCG GAT GGT TGG GGT TTG TCC-3’).

### Flow cytometry

24 h post activation, cells were labeled with anti-CD4/FITC (Affymetrix/eBioscience, San Diego, CA), anti-CD25/APC (Affymetrix/eBioscience, San Diego, CA) and anti-CD69/PE-Cy7 (BioLegend, San Diego, CA). After washing and resuspending the cells in FACS buffer, fluorescence was detected by a BD Accuri C6 flow cytometer and quantified by C-Flow software (BD Accuri, San Jose, CA).

### IL-2, IFNγ, TNFα, and GM-CSF ELISAs

Cell supernatants were collected from splenocytes and isolated CD4^+^ T cells 24 h post-activation. IL-2, IFNγ, TNFα, and GM-CSF were quantified from the supernatants using commercially available kits following the manufacturer’s protocols (BioLegend, San Diego, CA and Affymetrix/eBioscience, San Diego, CA). Absorbance was quantified at 450 nm using the Infinite M1000 Pro Microplate (Tecan, San Jose, CA).

### Quantification of c-fos DNA binding

4h post-activation, splenocytes were collected and nuclear protein was isolated using a commercially available kit (Active Motif, Carlsbad, CA). Nuclear protein was then quantified by Bradford Assay (Bio-Rad, Hercules, CA). 5 μg of nuclear protein was used to determine binding of c-fos to the AP-1 consensus binding site, as determined by quantification with an ELISA-based assay (TransAM kit, Active Motif, Carlsbad, CA).

### Statistical analysis

The mean ± SE was determined for each treatment group in individual experiments. Homogeneous data were analyzed by two-way parametric ANOVA. When significant differences were observed, the Holm-Sidak post hoc test was used to compare treatment groups using SigmaPlot 12.3 (Systat Software, San Jose, CA).

## Results

### Upregulation of Nrf2 target genes by ATO in murine splenocytes

**Fig 1:**
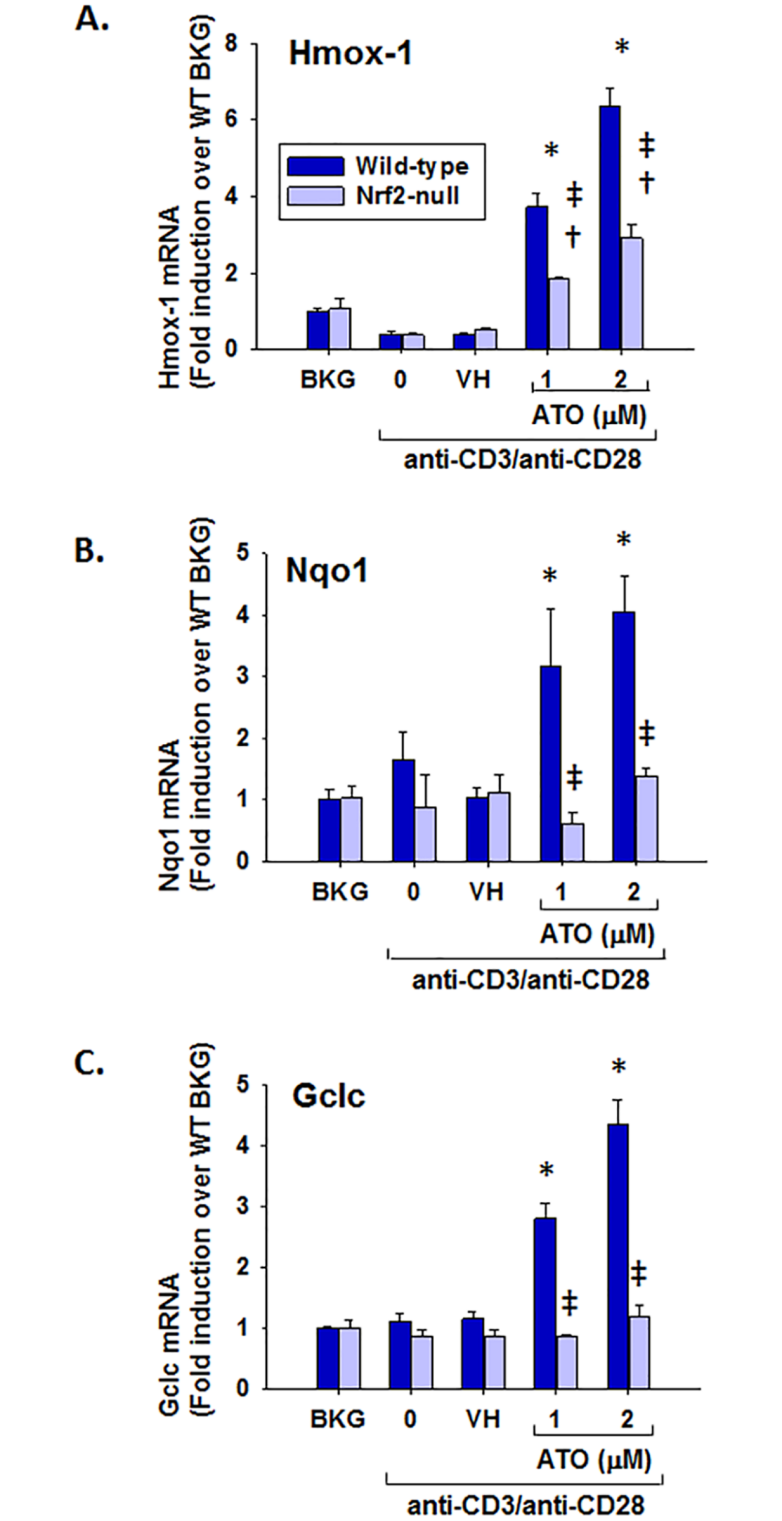
ATO upregulates the Nrf2 target genes Hmox-1, Nqo1, and Gclc in wild-type, but not Nrf2-null, splenocytes. Wild-type and Nrf2-null splenocytes were isolated and either left untreated (BKG) or treated with the vehicle (VH, PBS), 0 (activator alone), 1, or 2 μM ATO for 30 min. The cells were then either left unactivated (BKG) or activated with anti-CD3/anti-CD28 for 6 h. Real-time PCR was used to quantify the mRNA expression of (A) Hmox-1, (B) Nqo1, and (C) Gclc. * denotes p<0.05 compared to the wild-type VH group. † denotes p<0.05 compared to the Nrf2-null VH group. ‡ denotes p<0.05 between the wild-type and Nrf2-null genotypes.

Previous studies suggested that inorganic arsenic activates Nrf2 in numerous cell types. To determine the effect of ATO on Nrf2 activation in murine splenocytes, freshly isolated splenocytes were treated with clinically-relevant concentrations of ATO (1–2 μM) and activated with a T cell-specific activator, anti-CD3 and anti-CD28. Quantification of Nrf2 target gene expression revealed that Hmox-1, Nqo1, and Gclc mRNA levels were upregulated in the wild-type splenocytes when exposed to concentrations of ATO as low as 1 μM (Fig 1). However, this trend was significantly less pronounced, and often unobserved, in Nrf2-null splenocytes. Collectively, these data indicate that ATO upregulates Hmox-1, Nqo1, and Gclc through a largely Nrf2-dependent mechanism, demonstrating that Nrf2 is activated by ATO in murine splenocytes.

### Inhibition of IL-2, IFNγ, TNFα, and GM-CSF production by ATO

**Fig 2:**
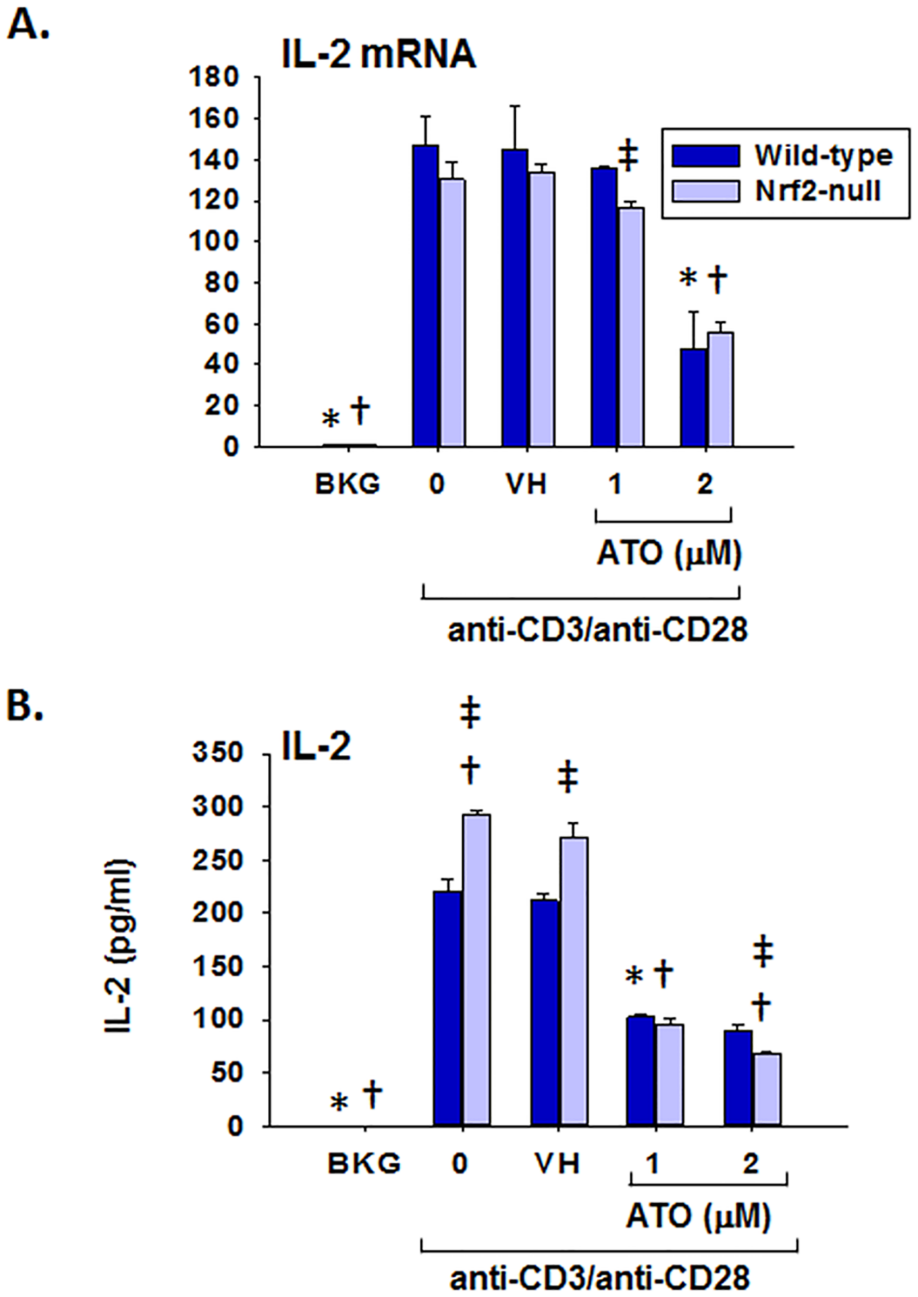
ATO markedly inhibits IL-2 production by anti-CD3/anti-CD28-activated splenocytes from wild-type and Nrf2-null mice. Wild-type and Nrf2-null splenocytes were isolated and either left untreated (BKG) or treated with vehicle (VH, PBS), 0 (activator alone), 1, or 2 μM ATO for 30 min. The cells were then either left unactivated (BKG) or activated with anti-CD3/anti-CD28 for (A) 6 h prior to quantification of IL-2 mRNA by real-time PCR, or (B) 24 h prior to quantification of IL-2 protein in cell supernatants by ELISA. * denotes p<0.05 compared to the wild-type VH group. † denotes p<0.05 compared to the Nrf2-null VH group. ‡ denotes p<0.05 between the wild-type and Nrf2-null genotypes.

**Fig 3:**
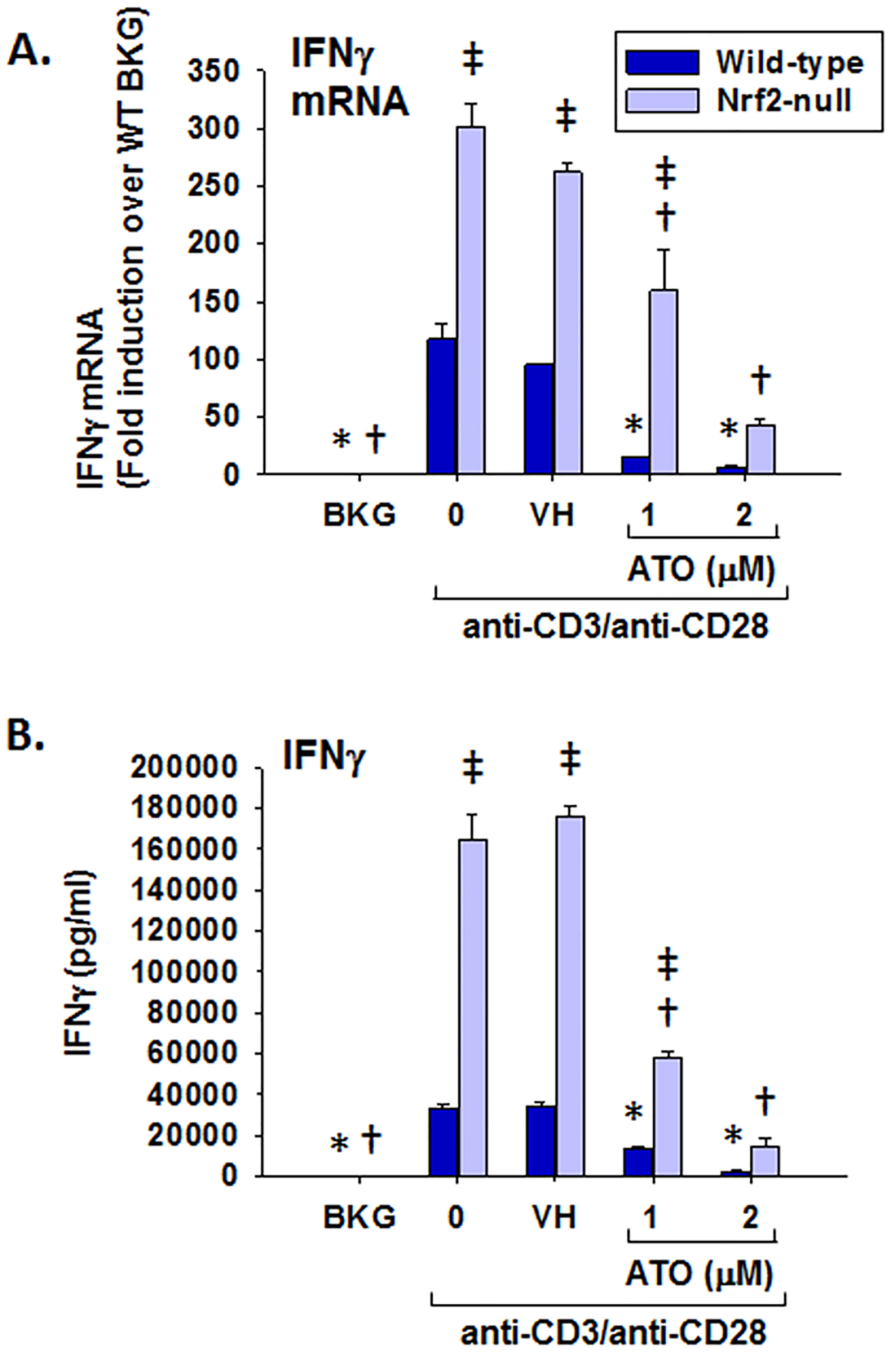
ATO markedly inhibits IFNγ production by anti-CD3/anti-CD28-activated splenocytes from wild-type and Nrf2-null mice. Wild-type and Nrf2-null splenocytes were isolated and either left untreated (BKG) or treated with the vehicle (VH, PBS), 0 (activator alone), 1, or 2 μM ATO for 30 min. The treatment groups were then either left unactivated (BKG) or activated with anti-CD3/anti-CD28 for (A) 6 h prior to quantification of IFNγ mRNA by real-time PCR, or (B) 24 h prior to quantification of IFNγ protein in cell supernatants by ELISA. * denotes p<0.05 compared to the wild-type VH group. † denotes p<0.05 compared to the Nrf2-null VH group. ‡ denotes p<0.05 between the wild-type and Nrf2-null genotypes.

**Fig 4:**
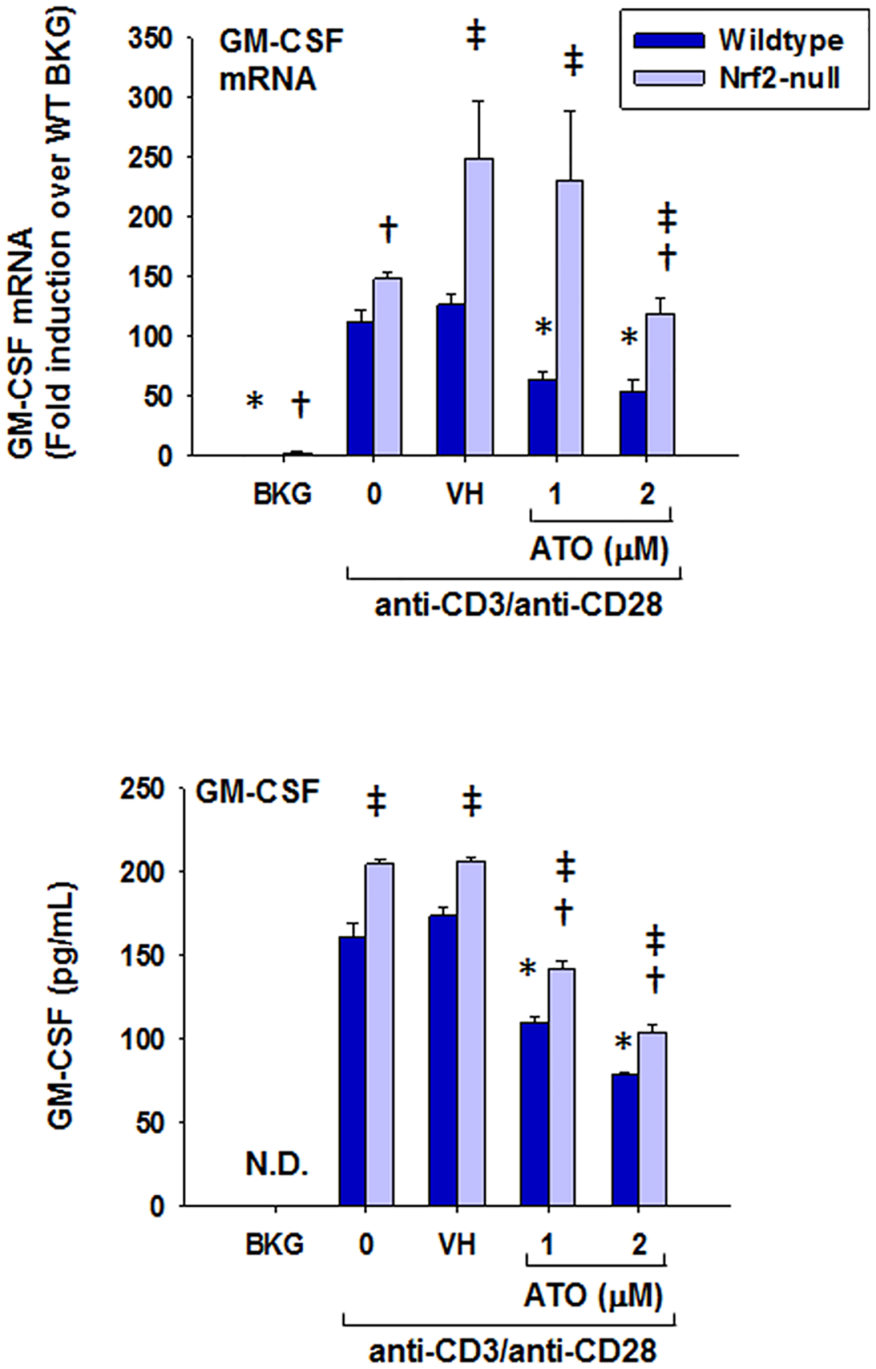
ATO markedly inhibits GM-CSF production by anti-CD3/anti-CD28-activated splenocytes from wild-type and Nrf2-null mice. Wild-type and Nrf2-null splenocytes were isolated and either left untreated (BKG) or treated with the vehicle (VH, PBS), 0 (activator alone), 1, or 2 μM ATO for 30 min. The treatment groups were then either left unactivated (BKG) or activated with anti-CD3/anti-CD28 for (A) 6 h prior to quantification of GM-CSF mRNA by real-time PCR, or (B) 24 h prior to quantification of GM-CSF protein in cell supernatants by ELISA. * denotes p<0.05 compared to the wild-type VH group. † denotes p<0.05 compared to the Nrf2-null VH group. ‡ denotes p<0.05 between the wild-type and Nrf2-null genotypes.

**Fig 5:**
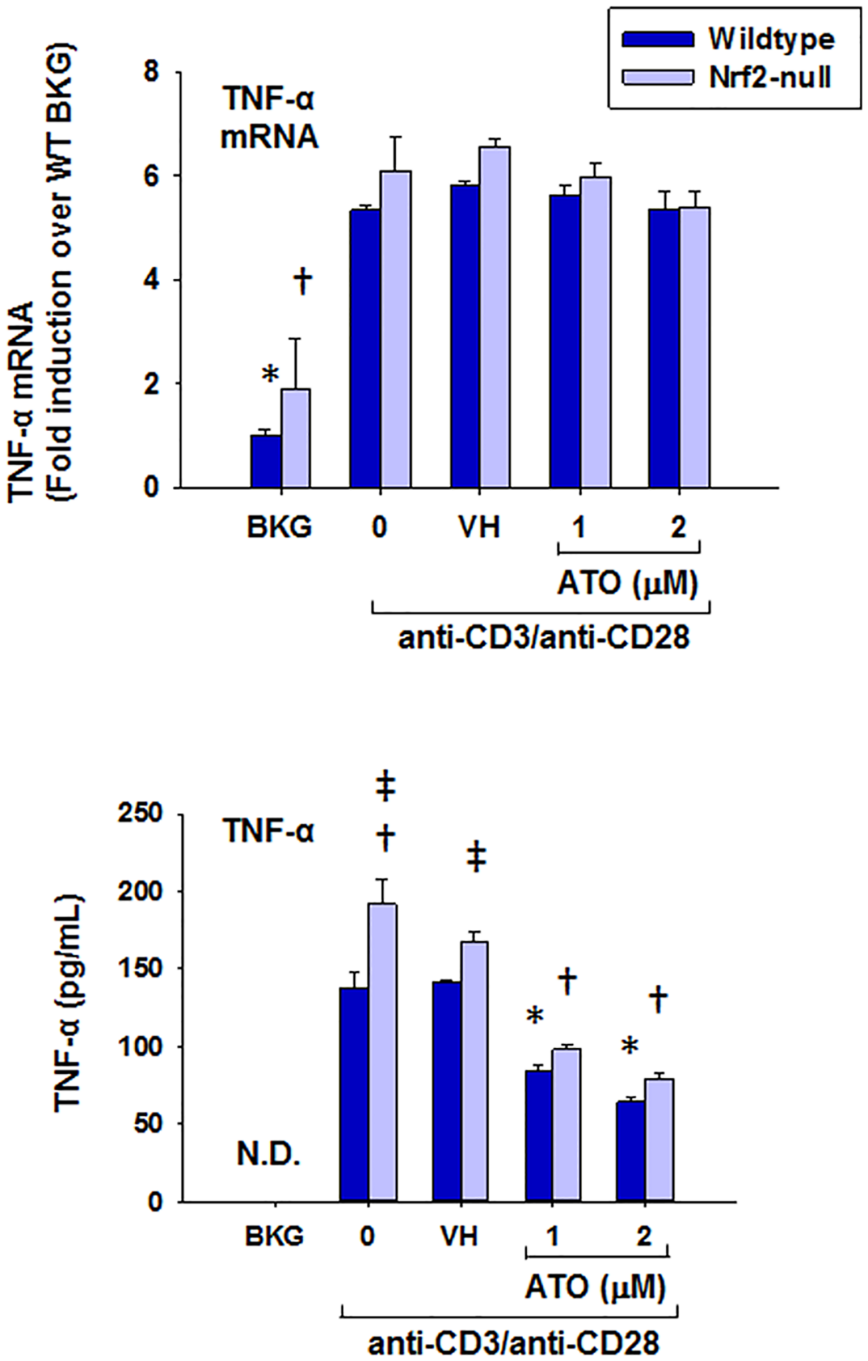
ATO markedly inhibits protein secretion, but not gene expression, of TNFα by anti-CD3/anti-CD28-activated splenocytes from wild-type and Nrf2-null mice. Wild-type and Nrf2-null splenocytes were isolated and either left untreated (BKG) or treated with the vehicle (VH, PBS), 0 (activator alone), 1, or 2 μM ATO for 30 min. The treatment groups were then either left unactivated (BKG) or activated with anti-CD3/anti-CD28 for (A) 6 h prior to quantification of TNFα mRNA by real-time PCR, or (B) 24 h prior to quantification of TNFα protein in cell supernatants by ELISA. * denotes p<0.05 compared to the wild-type VH group. † denotes p<0.05 compared to the Nrf2-null VH group. ‡ denotes p<0.05 between the wild-type and Nrf2-null genotypes.

**Fig 6:**
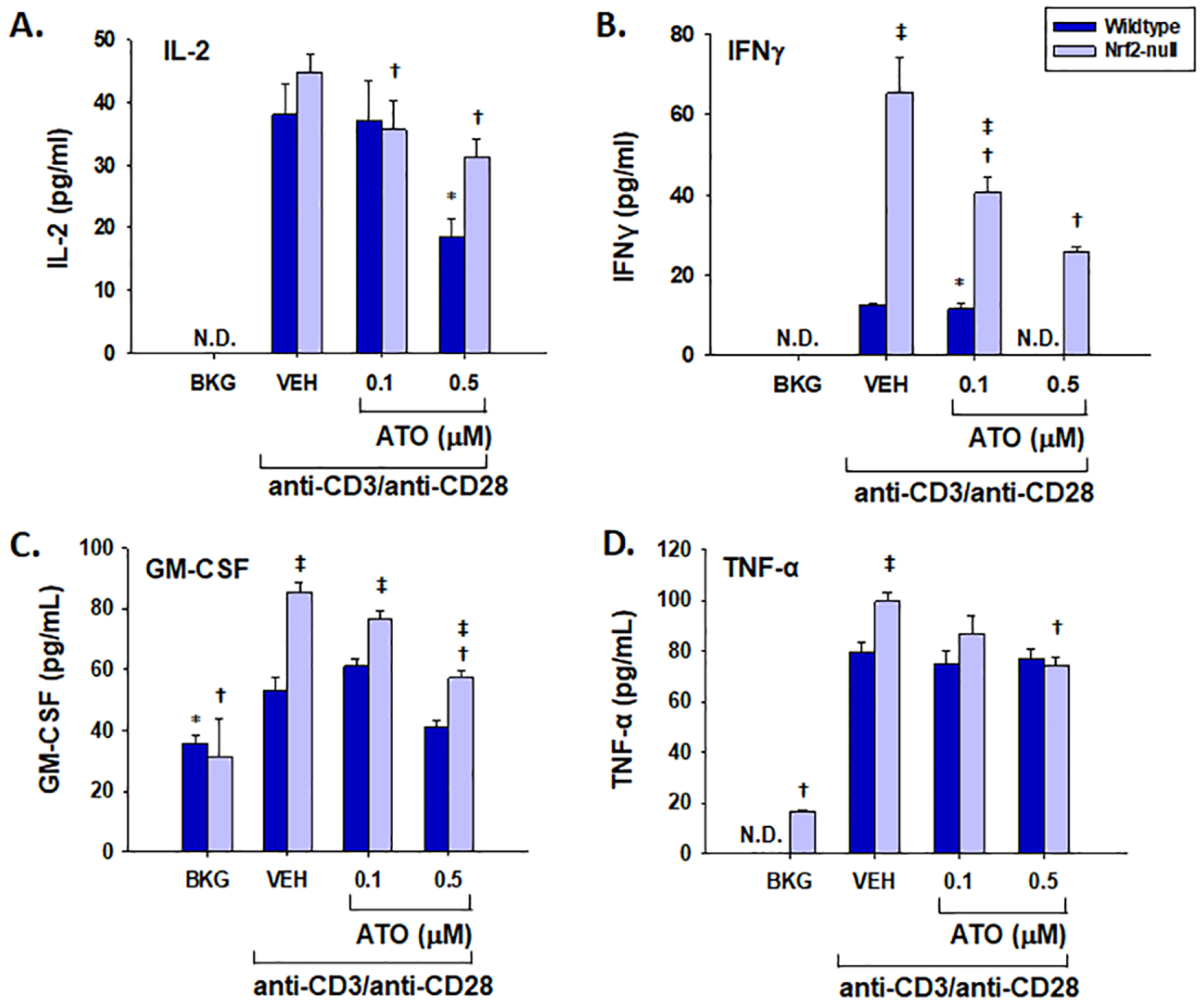
ATO inhibits cytokine production in isolated CD4^+^ T cells in a Nrf2-independent manner. Wild-type and Nrf2-null CD4^+^ T cells were isolated from splenocytes. They were left untreated (BKG) or treated with vehicle (VEH, PBS), 0.1 μM ATO, or 0.5 μM ATO for 30 min. The treatment groups were then either left unactivated (BKG) or activated with anti-CD3/anti-CD28 for 24 h prior to quantification of (A) IL-2, (B) IFNγ, (C) GM-CSF, and (D) TNFα protein in cell supernatants by ELISA. * denotes p<0.05 compared to the wild-type VH group. † denotes p<0.05 compared to the Nrf2-null VH group. ‡ denotes p<0.05 between the wild-type and Nrf2-null genotypes.

In addition to Nrf2 target gene expression, T cell immune parameters were also quantified. ATO treatment significantly inhibited IL-2 production by anti-CD3/anti-CD28-activated splenocytes at both the mRNA and protein levels (Fig 2). In addition, ATO inhibited induction of IFNγ and GM-CSF mRNA and protein in anti-CD3/anti-CD28-activated splenocytes (Figs 3 and 4). Notably, IFNγ production was significantly greater in splenocytes derived from Nrf2-null mice compared to wild-type, which is consistent with our previously published observations [31]. The same genotype effect appears to exist for GM-CSF in Nrf2-null mice which is also consistent with the literature [28]. Surprisingly, TNFα mRNA remained unaffected by treatment with ATO, though TNFα protein expression was inhibited in a similar fashion to the other measured cytokines (Fig 5). Interestingly, the inhibition of each cytokine’s production by ATO was observed in both the wild-type and the Nrf2-null splenocytes, suggesting that the effect of ATO on these cytokines is independent of Nrf2. Decreased production of IL-2, IFNγ by ATO treatment also occurred in activated CD4^+^ T cells; production of TNFα and GM-CSF was reduced in the Nrf2-null, but not wild-type, CD4^+^ cells. Reduced ATO concentrations were used in isolated CD4^+^ T cells due to increased toxicity compared with splenocytes (Fig 6).

### ATO does not alter CD25 and CD69 expression by activated splenocytes

**Fig 7:**
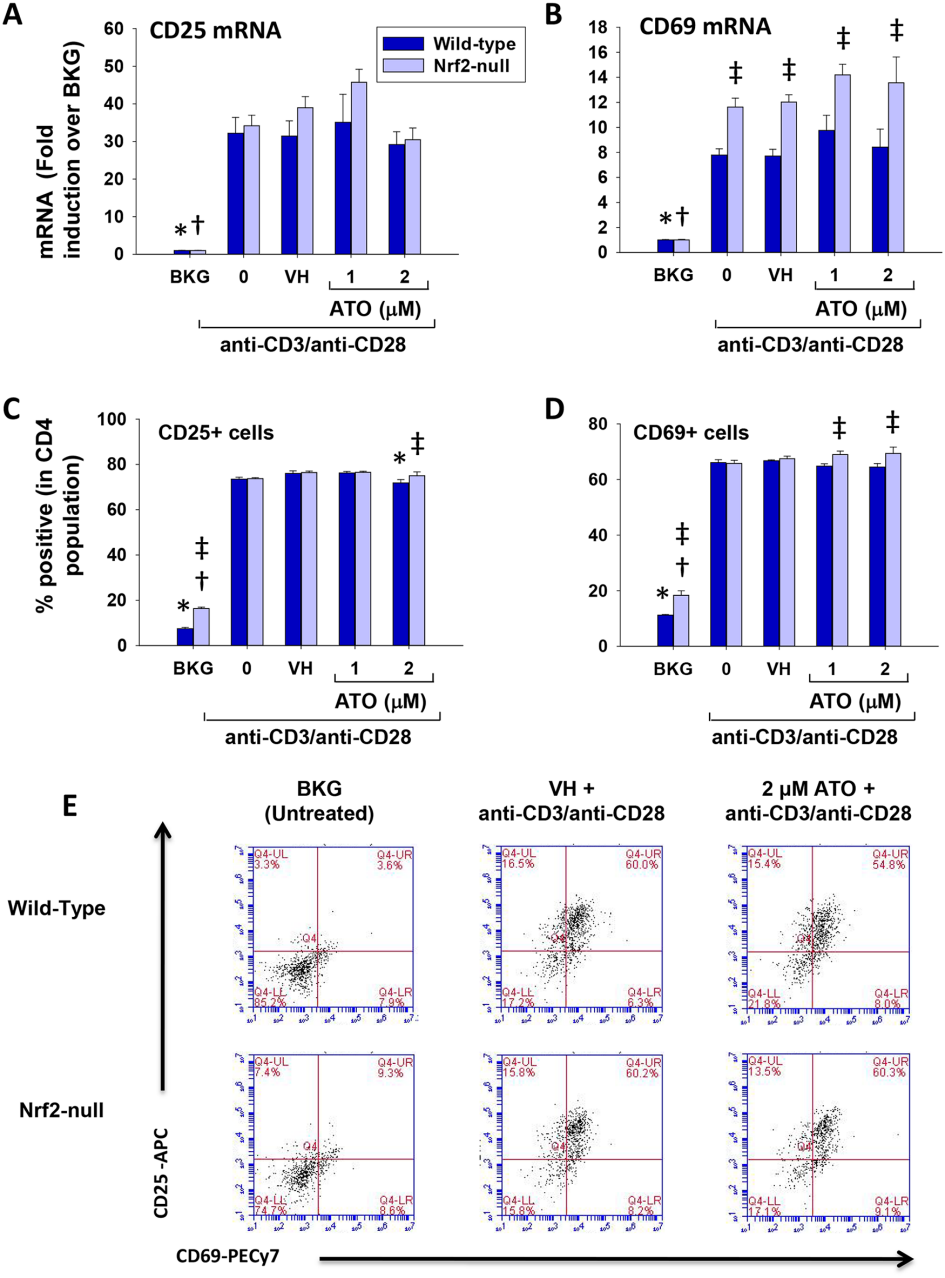
ATO does not affect expression of CD69 or CD25 in anti-CD3/anti-CD28-activated splenocytes. Wild-type and Nrf2-null splenocytes were isolated and either left untreated (BKG) or treated with the vehicle (VH, PBS), 0 (activator alone), 1, or 2 μM ATO for 30 min. The cells were then either left unactivated (BKG) or activated with anti-CD3/anti-CD28 for either (A-B) 6 h prior to quantification of (A) CD25 mRNA and (B) CD69 mRNA by real-time PCR or (C-E) 24 h for quantification of CD25+ and CD69+ cells by flow cytometry. The CD4+ T cells were gated on prior to analysis of CD25 and CD69 expression. (E) Representative dot plots of CD25 and CD69 cell surface protein expression in CD4 T cells. * denotes p<0.05 compared to the wild-type VH group. † denotes p<0.05 compared to the Nrf2-null VH group. ‡ denotes p<0.05 between the wild-type and Nrf2-null genotypes.

The suppression of cytokine production by ATO prompted us to investigate the induction of other genes that are upregulated during T cell activation. In contrast to the aforementioned cytokines, induction of CD25 and CD69 mRNA and protein expression was not impacted by ATO treatment in activated splenocytes from either wild-type or Nrf2-null mice (Fig 7). Overall, these data suggest that ATO has differential effects on early events following T cell activation.

### Inhibition of AP-1 binding by ATO in activated splenocytes

**Fig 8:**
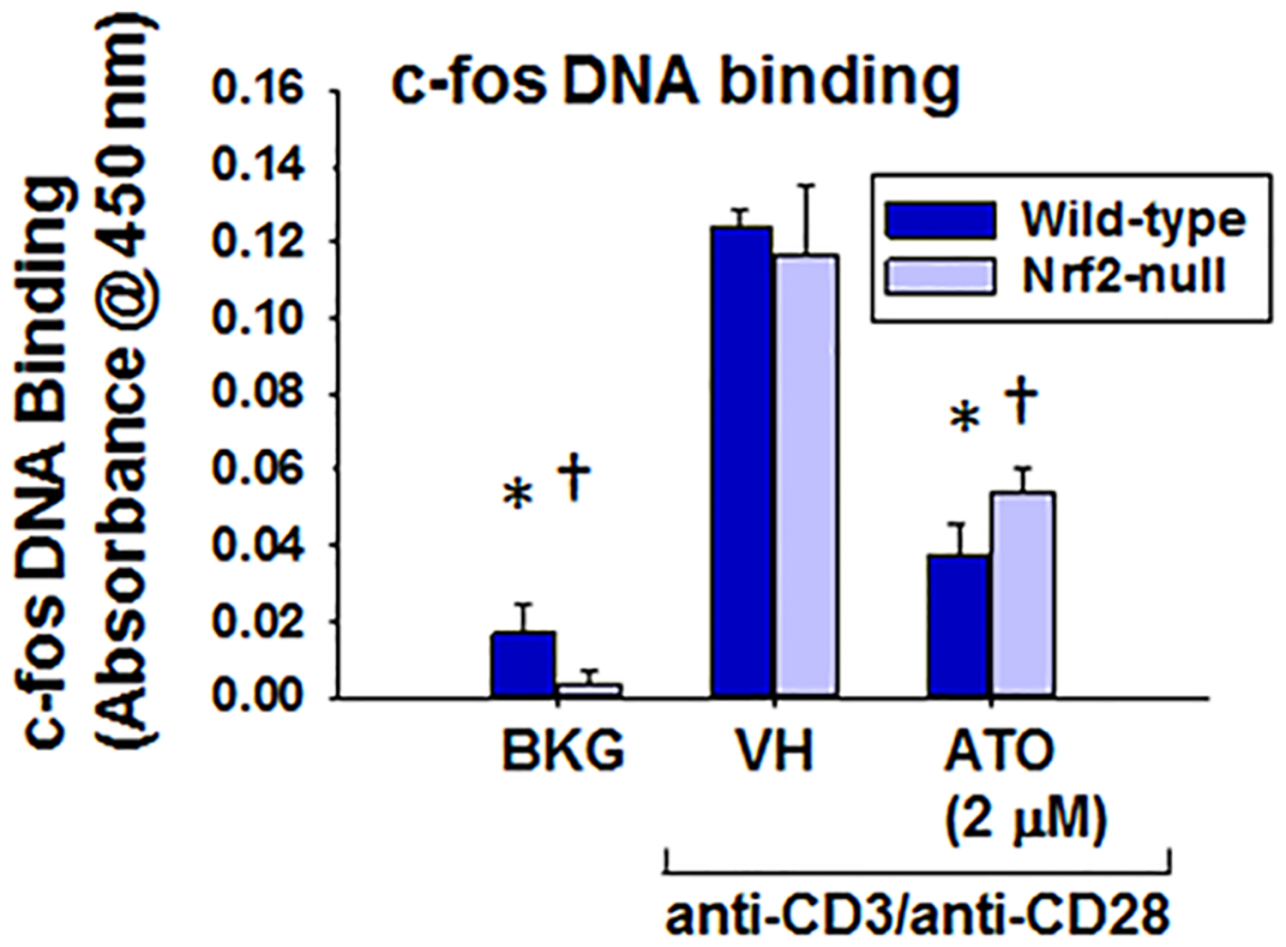
ATO decreased c-fos DNA binding in anti-CD3/anti-CD28-activated splenocytes from wild-type and Nrf2-null mice. Wild-type and Nrf2-null splenocytes were isolated and either left untreated (BKG) or treated with the vehicle (VH, PBS) or 2 μM ATO for 30 min. The cells were either then left unactivated (BKG) or activated with anti-CD3/anti-CD28 for 4 h after which cells were harvested and nuclear protein extracted. Binding of c-fos to the AP-1 consensus binding site was quantified colorimetrically using an ELISA-based assay. * denotes p<0.05 compared to the wild-type VH group. † denotes p<0.05 compared to the Nrf2-null VH group.

Because the effect of ATO on cytokine expression appeared to be Nrf2-independent, we considered alternative mechanisms. We identified the AP-1 and NFκB transcription factors as key to promoting the expression of all the cytokines quantified in this study. Previous studies established that NFκB is also critical for the induction of CD25, while regulation of CD69 and CD25 by AP-1 is more complex and may involve other transcription factors [34–39]. Since we observed no effects of ATO on CD25 or CD69 expression, we focused on AP-1 as a potential mechanism. Accordingly, the effect of ATO on c-fos DNA binding was evaluated. Consistent with our cytokine data, activation of isolated splenocytes with anti-CD3/anti-CD28 markedly decreased c-fos binding to the AP-1 consensus binding site in a Nrf2-independent fashion (Fig 8). Taken together, these data suggest that the inhibition of cytokine induction by ATO in anti-CD3/anti-CD28-activated splenocytes may be due to inhibition of c-fos activation, independent of Nrf2.

## Discussion

It has been previously shown that chronic exposure to inorganic arsenic has immunosuppressive effects that correlate with altered cytokine production [6,7,10]. In addition, inorganic arsenic has been shown to activate the stress-induced transcription factor Nrf2. Furthermore, studies by our lab and others have established that Nrf2 alters cytokine production by activated T cells and other leukocytes [16,17,40]. Thus, this study aimed at determining whether ATO affects early events following T cell activation, including cytokine production. In addition, we sought to determine whether Nrf2 is activated by ATO in primary mouse splenocytes and to identify the role of Nrf2 in the effects of ATO on the early events following T cell activation.

The current study demonstrates that ATO activates Nrf2 in murine splenocytes, as evidenced by induction of Nrf2 target gene expression in wild-type, but not Nrf2-null splenocytes. Whereas ATO markedly inhibits the upregulation of IL-2, IFNγ TNFα, and GM-CSF in anti-CD3/anti-CD28-activated splenocytes, it has little effect on the induction of CD25 and CD69, cell surface molecules that are rapidly upregulated during T cell activation. Interestingly, ATO inhibited IL-2, IFNγ, and GM-CSF, but not TNFα, mRNA production in both wild-type and Nrf2-null splenocytes, whereas protein secretion of IL-2, IFNγ, GM-CSF, and TNFα, was inhibited by ATO exposure. These results suggest that the inhibition of cytokine secretion by ATP occurs independently of Nrf2 and further suggest post-transcriptional modulation of TNFα by ATO. In support of this, the literature has shown TNFα to be both transcriptionally and post-transcriptionally regulated by various mechanisms, including microRNA and metabolic state, which could explain why we observed an alteration on TNFα protein levels, but not mRNA [41–44]. In addition, we found that ATO significantly inhibits c-fos binding to the AP-1 consensus binding site in a Nrf2-independent manner, which correlates with the decrease in cytokine expression. Collectively, the present study is the first to demonstrate that ATO has differential effects on T cell activation in which CD25/CD69 expression is unaffected, but IL-2, IFNγ, TNFα, and GM-CSF production are markedly decreased. Furthermore, the inhibition of cytokine production by ATO is independent of Nrf2 and correlates with decreased c-fos DNA binding, suggesting the effect may be due to impaired AP-1 activity.

In agreement with splenocytes, isolated CD4^+^ T cells showed no changes in CD25 or CD69 expression (data not shown) along with decreased IL-2 and IFNγ secretion that was independent of Nrf2. Interestingly, secretion of TNFα and GM-CSF by wild-type CD4^+^ cells were unaffected by ATO, while CD4^+^ cells from Nrf2-null animals remained sensitive. Our results indicate that CD4^+^ T cells are directly affected by ATO exposure, but results differ from the splenocyte preparation depending on cytokine, indicating that antigen-presenting cells have some effect on cytokine secretion in the presence of ATO.

CD25, the alpha chain of the IL-2 cell receptor, and CD69, a C-type lectin protein, are rapidly upregulated during T cell activation. As such, they are useful markers of early T cell activation. Our data indicate that at sub-cytotoxic levels of ATO (≤2μM for splenocytes, ≤0.5μM for CD4^+^ T cells), neither CD25 nor CD69 expression is significantly altered in anti-CD3/anti-CD28-activated splenocytes from either wild-type or Nrf2-null mice. Whereas previously published data indicate that sodium arsenite decreases CD69 surface expression in PBMCs isolated from C57BL/6 mice and humans, the current study demonstrates that ATO does not affect CD69 expression on splenic CD4 T cells [45,46]. Overall, these studies suggest that trivalent arsenic may have differential effects on CD69 expression, which may be dependent on the specific congener of trivalent arsenic.

The current study also demonstrates that ATO markedly inhibits early production of IL-2, IFNγ, TNFα, and GM-CSF in anti-CD3/anti-CD28-activated splenocytes, as well as IL-2 and IFNγ in isolated CD4^+^ T cells, which is consistent with previous studies [7,12,45,47,48]. By utilizing Nrf2 knockout mice in which Nrf2 expression is completely ablated, we found that lack of Nrf2 expression did not rescue the inhibition of cytokine production by ATO. Similar to our findings, a previous study conducted in human T cells isolated from PBMCs revealed that while inorganic arsenic exposure (2μM) decreased the expression of certain cytokines—including IL-2, IFNγ, and TNFα –partial silencing of Nrf2 (~45% knockdown) by transient siRNA transfection did not rescue the inhibition [49]. Collectively, these studies demonstrate that inhibition of IL-2, IFNγ, TNFα, and GM-CSF induction by inorganic arsenic in activated T cells occurs independently of Nrf2.

T cell activation results in the initiation of multiple kinase cascades that lead to the activation of several transcription factors that subsequently upregulate cytokine production [50,51]. One such transcription factor is AP-1, which is composed of a c-fos/c-jun heterodimer, and is a known regulator of IL-2, IFNγ, TNFα, and GM-CSF transcription [52–56]. The present study shows that ATO impairs c-fos binding to the AP-1 binding site. There are conflicting reports with respect to the effect of ATO on c-fos expression and activity. Whereas several studies show that ATO upregulates c-fos mRNA expression in a number of different cell types, ATO has also been shown to decrease c-fos expression in HL-60 cells, an immune cell line [57]. Our results are consistent with the latter report. The reason for the differential effects of ATO on c-fos expression in different cell types is not yet clear. Although Nrf2 can directly interact with AP-1 family members and thereby affect AP-1 activity, the inhibition of c-fos by ATO was observed in both wild-type and Nrf2-null splenocytes, suggesting the effect is independent of Nrf2 [58]. The decrease in c-fos DNA binding correlates with the reduction in cytokine production and thus is a likely mechanism for the observed decrease in IL-2, IFNγ, TNFα, and GM-CSF induction by ATO in activated T cells.

Altogether, this study suggests that while Nrf2 is activated by ATO in murine splenocytes stimulated with the T cell activator anti-CD3/anti-CD28, the effects of ATO on cytokine production are independent of Nrf2 activation. Furthermore, this study demonstrates that ATO has differential effects on the early events of T cell activation in which it markedly inhibits production of IL-2, IFNγ, TNFα, and GM-CSF but does not affect induction of CD69 and CD25. Lastly, this study is the first to show that the inhibition of IL-2, IFNγ, TNFα, and GM-CSF production by ATO correlates with decreased c-fos DNA binding, which suggests the effect of ATO on cytokine production is due to impaired AP-1 activity.
